# Maternal Protein Restriction Alters the Renal *Ptger1* DNA Methylation State in SHRSP Offspring

**DOI:** 10.3390/nu10101436

**Published:** 2018-10-05

**Authors:** Moe Miyoshi, Masayuki Sato, Kenji Saito, Lila Otani, Katsuhiko Shirahige, Fumihito Miura, Takashi Ito, Huijuan Jia, Hisanori Kato

**Affiliations:** 1 Health Nutrition, Graduate School of Agricultural and Life Sciences, The University of Tokyo, Tokyo 1138657, Japan; monet344myo@gmail.com (M.M.); sato-mas@lion.co.jp (M.S.); kkkj774@yahoo.co.jp (K.S.); nf_lila@yahoo.co.jp (L.O.); ginajhj@gmail.com (H.J.); 2 Research Center for Epigenetic Disease, Institute of Molecular and Cellular Biosciences, The University of Tokyo, Tokyo 1130032, Japan; kshirahi@gmail.com; 3 Department of Biochemistry, Kyushu University Graduate School of Medical Sciences, Kyushu University, Fukuoka 8128582, Japan; fumihito@med.kyushu-u.ac.jp (F.M.); tito@med.kyushu-u.ac.jp (T.I.)

**Keywords:** maternal protein restriction, salt sensitivity, DNA methylation

## Abstract

We previously reported that maternal protein restriction (LP) during pregnancy increases salt sensitivity in offspring using the Stroke-Prone Spontaneously Hypertensive Rat (SHRSP). In the present study, we focus on DNA methylation profiles of prostaglandin E receptor 1 gene (*ptger1*), which is known to be associated with hypertension. We evaluated the *ptger1* DNA methylation status via bisulfite sequencing, and analyzed the expression of *ptger1*-related genes. The results of these analyses showed that, compared to controls, the LP-S offspring exhibited both marked *ptger1* hypermethylation, and significantly increased *ptger1* expression. Moreover, they also exhibited significantly decreased expression of the downstream gene epithelial Na^+^ channel alpha (*enacα*). Interestingly, LP offspring that were provided with a standard water drinking supply (W) also exhibited increased *ptger1* methylation and expression. Together, these results suggest that maternal protein restriction during pregnancy modulates the renal *ptger1* DNA methylation state in SHRSP offspring, and thereby likely mediates *ptger1* and *enacα* gene expression to induce salt sensitivity.

## 1. Introduction

In recent years, the concept that the fetal nutritional state affects the lifetime risk of pathophysiological processes associated with chronic (including especially non-communicable) diseases, (i.e., the ‘Fetal Origins of Adult Disease (FOAD)’, and ‘Developmental Origins of Health and Disease (DOHaD)’ concepts originally proposed by Barker in the 1990s [1,2,3]), has become widely accepted. For example, several studies have demonstrated that low birth weight caused by maternal undernutrition is strongly associated with childhood obesity, diabetes, and cardiovascular disorders [4,5,6,7].

Epigenetic modifications are chemical changes to DNA and/or histones that regulate gene expression without altering the DNA sequence. Previous studies have shown that DNA methylation changes tend to be retained in chromatin across generations [8]; thus, they have been proposed as possible key mechanisms that underlie DOHaD [9,10,11].

The Stroke-Prone Spontaneously Hypertensive rat (SHRSP) naturally develops stroke as a result of harboring a heredity factor that causes hypertension, and has accordingly been widely used as a research model for cardiovascular diseases. In our previous study using the SHRSP, protein restriction during pregnancy significantly increased the rate of salt-sensitive hypertension and early death among offspring that were provided with a 1% saline drinking solution, but not among those that were administered tap water [12,13]. Similarly, the production of aldosterone (a key factor in the renin-angiotensin-aldosterone system (RAAS)) was increased, and type II angiotensin receptor protein levels in the kidney and adrenal gland were markedly affected in the offspring of dams that underwent protein restriction during pregnancy [13,14]. Therefore, in the present study, we hypothesized that epigenetic changes may modulate salt sensitivity after maternal protein restriction, and accordingly attempted to identify novel target genes via a DNA methylation analysis.

## 2. Materials and Methods

### 2.1. Ethics Statement and Animal Conditions

The present study was approved, and conducted in strict accordance with the guidelines stipulated by the Animal Usage Committee of the Graduate School of Agricultural and Life Sciences, at The University of Tokyo, (Approval No. P09-376). Rats were maintained at 22 ± 1 °C, in 60% ± 5% humidity, and in a 12 h light (8:00 to 20:00)/dark cycle.

### 2.2. Animal Experiment 1: Water-Drinking Offspring

Eight-week-old male and female SHRSP/Izm rats were obtained from the Disease Model Cooperative Research Association (Kyoto, Japan). After acclimatizing to the stipulated environmental conditions for 1 week, virgin female SHRSP rats (body weight, 169–183 g; blood pressure, 173–186 mmHg) were mated to a single stud male (body weight, 241–260 g; blood pressure, 175–187 mmHg). Pregnancy was confirmed by the presence of a semen plug. Pregnant dams were randomly allocated to be fed a 20% (CN) or 9% casein diet (LP) during pregnancy (Table 1). After delivery, all dams were fed a commercial diet (MF diet; Oriental Yeast, Tokyo, Japan). At the fourth postnatal week, male pups were separated from the dams, and provided with ad libitum access to drinking water (W) and a commercial diet. Finally, 12-week-old male (CN-W or LP-W) offspring were culled, and their kidney, brain, and heart samples were collected.

### 2.3. Animal Experiment 2: Salt-Loading Offspring

Virgin female SHRSP rats (body weight, 152–165 g; blood pressure, 161–176 mmHg) were mated to a stud male (body weight, 217–227 g; blood pressure, 153–173 mmHg). The rats were maintained as described in Animal Experiment 1 during pregnancy and lactation period. Male offspring were separated from the dams at the fourth postnatal week provided with ad libitum access to drinking water and commercial diet, and then they were administered a 1% saline solution (S) during weeks 10–12 after birth (CN-S or LP-S).

### 2.4. Blood Pressure Measurement

Blood pressure was measured as described in our previous report [12]. Briefly, blood pressure measurements were taken from the tail (BP-98A; Softron, Tokyo, Japan) of offspring at 10, 11, and 12 weeks after birth, without sedation.

### 2.5. Biochemical Analysis

Urine samples were collected over a 4-day period from 11-week-old offspring, and the urinary albumin excretion level was measured using a Rat Albumin ELISA kit (AKRAL-120; Shibayagi, Gunma, Japan). Plasma was collected from 12-week-old offspring, and the aldosterone concentration was measured using a DetectX^®^ Aldosterone Enzyme Immunoassay kit (Arbor Assays, Michigan, MI, USA).

### 2.6. Quantitative Real-Time PCR

The total RNA from kidney samples was extracted using a NucleoSpin^®^ TriPrep kit (Takara Bio, Tokyo, Japan), and reverse transcribed to cDNA using PrimeScript^TM^ RT Master Mix (Perfect Real Time) (Takara Bio, Tokyo, Japan). Gene segments were amplified from the synthesized cDNA using the Thermal Cycler Dice Real Time System TP800 (Takara Bio, Tokyo, Japan), SYBR^®^ Premix Ex Taq™ (Tli RNaseH Plus) (Takara Bio, Tokyo, Japan), and appropriate primers (Supplementary Information (SI) Table S1). mRNA levels were normalized to those of *18S* rRNA, and expressed as fold-change values.

### 2.7. Analysis of DNA Methylation Profile

To determine the target gene for DNA methylation analysis, we conducted an exploratory genome-wide methylation (methylome) analysis (*n* = 1) and found that one of the genes located within/adjacent to the differentially methylated regions identified in the salt-sensitized offspring was the prostaglandin E receptor 1 gene (*ptger1*).

Then we focused on the methylation status of CpG sites (CpGs) within the prostaglandin E receptor 1 (*ptger1*) gene (region from transcription initiation site; +1315 to +1614, 300 bp) and examined it via bisulfite sequencing. Briefly, renal genomic DNA was bisulfite-converted using a BisulFlash^TM^ DNA Modification Kit (Epigentek, Farmingdale, NY, USA), and amplified using EpiTaq^TM^ HS (for bisulfite-treated DNA) (Takara Bio, Tokyo, Japan) and *ptger1* primers (sense, 5′-AATATATTTGTGGTGTTGTTAATAGG-3′; antisense, 5′-ACCAAAAAAAACCATACAACC-3′). The amplified PCR products were separated via 1% Agarose-ME (Nacalai Tesque, Inc., Tokyo, Japan) gel electrophoresis, and purified using the Wizard SV Gel and PCR Clean-Up system (Promega, Madison, WI, USA). They were then ligated into the pGEM-T easy vector (Promega, Madison, WI, USA), and transformed into *Escheria coli* DH5α competent cells (Sigma-Aldrich, Saint Louis, MO, USA). Plasmid DNA was isolated using a Gene Elute^TM^ Plasmid MiniPrep kit (Sigma-Aldrich, Saint Louis, MO, USA). Clones were commercially sequenced by Eurofins Genomics (Tokyo, Japan), and sequencing data were analyzed using Quantification tool for Methylation Analysis software (http://quma.cdb.riken.jp/) (Last updated at Jul 19 00:30:00 JST 2014) (RIKEN Center for Developmental Biology, Kobe, Japan).

### 2.8. Statistical Analysis

Values were expressed as the mean ± standard error. The statistical significance of data produced by Animal Experiments 1 and 2 was assessed using a one-way analysis of variance (ANOVA), followed by a Student’s *t*-test. A *p* value < 0.05 was considered to indicate statistical significance.

## 3. Results

### 3.1. Blood Pressure of Offspring from 10 to 12 Weeks after Birth

**Figure 1:**
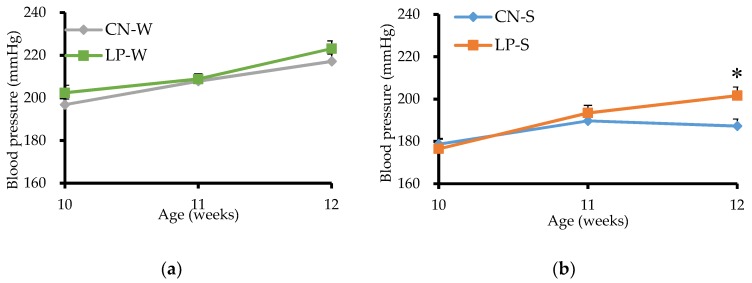
Effect of low protein intake during pregnancy on the blood pressure of offspring. Blood pressure changes from 10 to 12 weeks after birth in (**a**) CN-W and LP-W (Animal Experiment 1), and (**b**) CN-S and LP-S (Animal experiment 2) offspring. Values are expressed as the mean ± standard error (*n* = 7–8). * *p* < 0.05 vs. the CN-S group, according to a Student’s *t*-test. Offspring were exposed to either an intrauterine environment of 20% (CN)-feeding mothers or that of 9% (LP)-Casein diet-feeding mothers, and provided with either water (W) or 1% saline drinking supply (S).

No significant differences were observed between the liquid consumption of the CN-W and CN-S compared to the LP-W and LP-S offspring, respectively, between 10 and 12 weeks after birth (SI Tables S2 and S3). Similarly, the blood pressures of the CN-W and LP-W offspring were not significantly different between weeks 10 to 12 after birth (Figure 1a). In contrast, the blood pressure of the LP-S offspring was significantly elevated compared to that of the CN-S offspring at 12 weeks-of-age (Figure 1b).

### 3.2. Offspring Organ Weight, and Urinary Albumin Excretion, and Plasma Aldosterone Levels at 12 Weeks after Birth

The induced changes to maternal protein intake did not affect the final body weight of the offspring (SI Tables S2 and S3). Likewise, all offspring administered drinking water exhibited similar kidney, brain, and heart weights (SI Table S2). In contrast, while the kidney and brain weights exhibited by the two groups of offspring provided with the saline drinking solution were equivalent, the heart weights exhibited by the LP-S offspring were significantly higher than those of the CN-S offspring, (SI Table S3).

**Figure 2:**
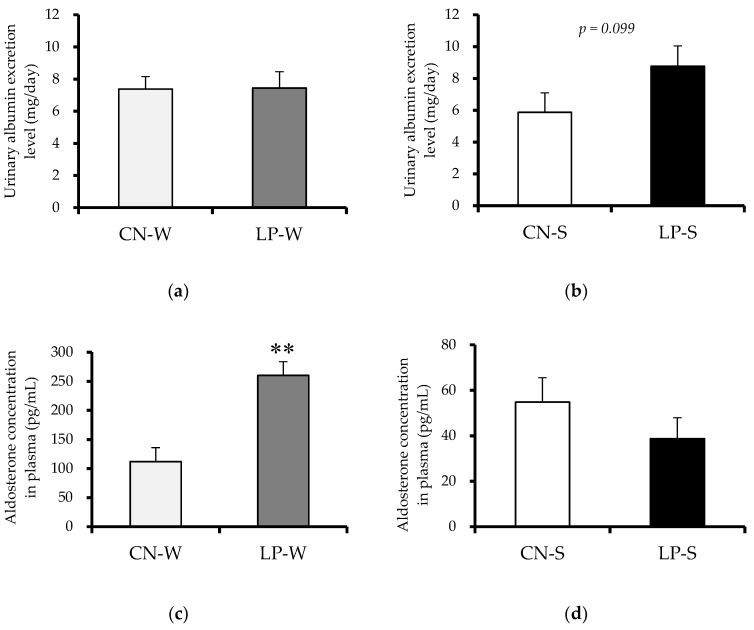
Effect of low protein intake during pregnancy on the exhibition of renal damage markers by offspring at 12 weeks after birth. (**a**) Offspring urinary albumin excretion levels (Animal Experiment 1). (**b**) Offspring urinary albumin excretion levels (Animal Experiment 2). (**c**) Aldosterone concentration levels in offspring plasma (Animal Experiment 1). (**d**) Aldosterone concentration levels in offspring plasma (Animal Experiment 2). Values are expressed as the mean ± standard error (*n* = 7–8). ** *p* < 0.01 vs. the CN-W group according to a Student’s *t*-test. Offspring were exposed to either an intrauterine environment of 20% (CN)-feeding mothers or that of 9% (LP)-Casein diet-feeding mothers, and provided with either water (W) or 1% saline drinking supply (S).

No significant difference in urinary volume was observed between the CN-W and LP-W, nor the CN-S and LP-S offspring (SI Tables S2 and S3). However, the urinary albumin excretion level, which is a biomarker for acute kidney injury, was similar in the CN-W and LP-W offspring (Figure 2a), but tended to be higher in the LP-S than the CN-S offspring (Figure 2b). Conversely, while the plasma levels of the pivotal blood pressure regulator aldosterone were similar in the CN-S and LP-S offspring (Figure 2d), they were significantly increased in the LP-W compared to the CN-W offspring (Figure 2c).

### 3.3. DNA Methylation Status in the Ptger1 Gene Region in Offspring Kidney Samples

First of all, we determined to focus on *ptger1* gene by an exploratory methylome analysis, since *ptger1* encodes the EP1 subtype of the prostaglandin E2 (PGE2) receptor, which is known to both mediate algesia, and regulate blood pressure.

**Figure 3:**
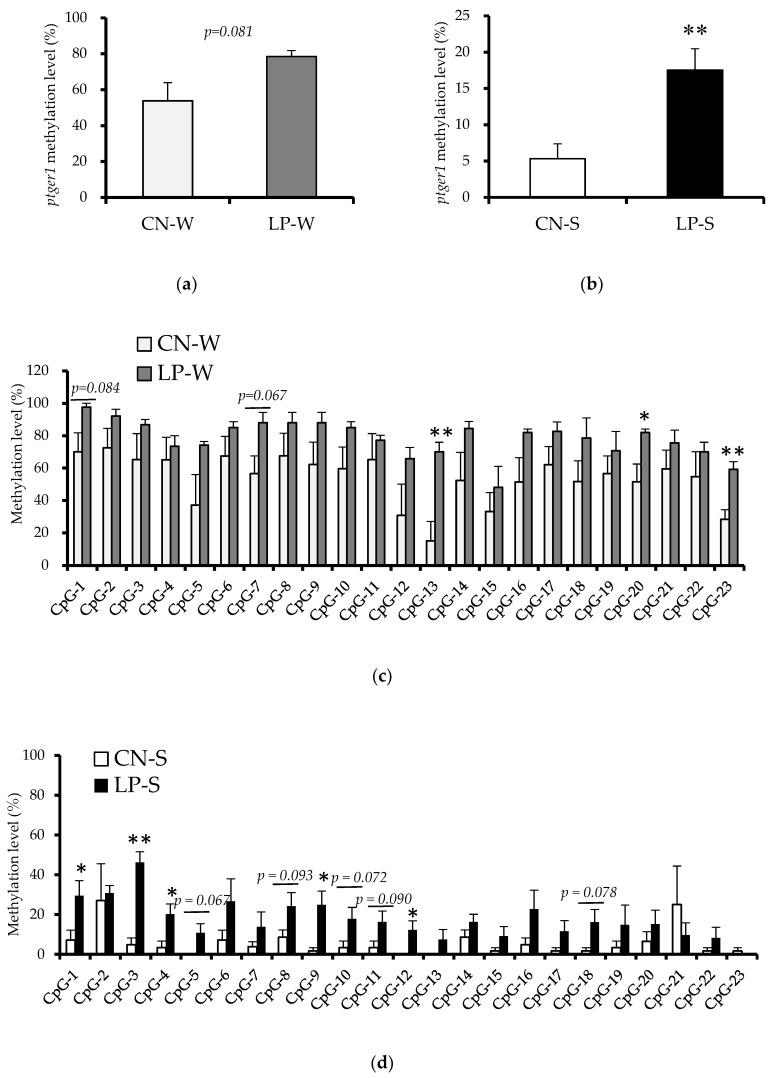
DNA methylation levels in the transcribed region of the *ptger1* gene in offspring kidney samples. (**a**) The *ptger1* total methylation level (Animal Experiment 1). (**b**) The *ptger1* total methylation level (Animal Experiment 2). (**c**) Methylation levels at 23 *ptger1* CpG sites (Animal Experiment 1). (**d**) Methylation levels at 23 *ptger1* CpG sites (Animal experiment 2). Values are expressed as the mean ± standard error. ** *p* < 0.01, * *p* < 0.05 vs. the CN-W or CN-S offspring according to a Student’s *t*-test. Offspring were exposed to either an intrauterine environment of 20% (CN)-feeding mothers or that of 9% (LP)-Casein diet-feeding mothers, and provided with either water (W) or 1% saline drinking supply (S).CN-W, *n* = 3; LP-W, *n* = 3; CN-S, *n* = 5; LP-S, *n* = 6.

Next all four groups of offspring (CN-W, *n* = 3; LP-W, *n* = 3; CN-S, *n* = 5; LP-S, *n* = 6) were subjected to bisulfite sequencing to investigate the DNA methylation status of the transcribed *ptger1* region (region from transcription initiation site; +1315 to +1614), which contains 23 CpGs. The overall DNA methylation levels in this region were shown to be higher in the LP-S, and to a lesser extent in the LP-W, than in the CN-S and CN-W offspring, respectively (Figure 3a,b, SI Figure S1). Three CpGs (CpG-13, CpG-20, and CpG-23) were significantly hypermethylated, and two others (CpG-1, and CpG-7) tended to be hypermethylated in the LP-W compared to the CN-W offspring (Fig. 3c). Similarly, five CpGs (CpG-1, CpG-3, CpG-4, CpG-9, and CpG-12) were significantly hypermethylated, and another five (CpG-5, CpG-8, CpG-10, CpG-11, CpG-18) tended to be hypermethylated in the LP-S compared to the CN-S offspring (Figure 3d).

### 3.4. Offspring Renal Expression Of Ptger1-Related Genes

**Figure 4:**
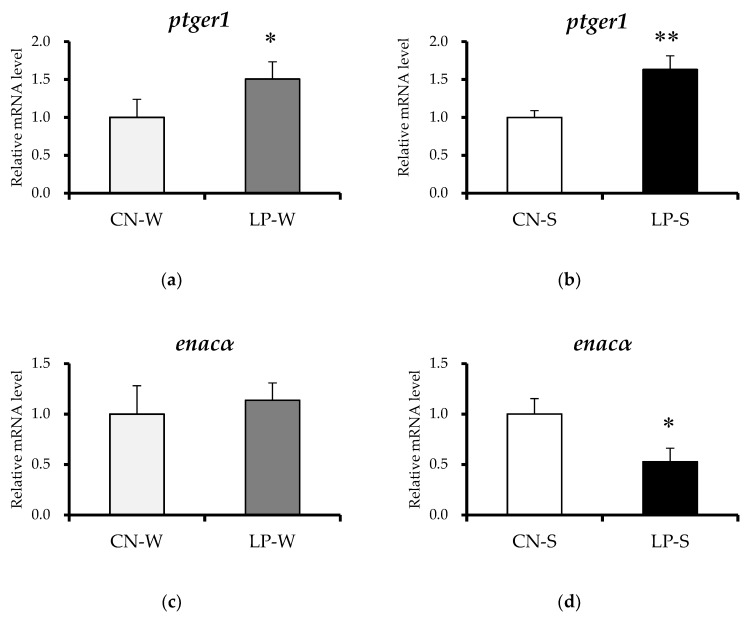
Relative renal expression of *ptger1* and *enacα* mRNA. (**a**) *ptger1* mRNA expression levels (Animal experiment 1). (**b**) *ptger1* mRNA expression levels (Animal Experiment 2). (**c**) *enacα* mRNA expression levels (Animal Experiment 1). (**d**) *enacα* mRNA expression levels (Animal Experiment 2). Values are expressed as the mean ± standard error (*n* = 7–8). ** *p* < 0.01, * *p* < 0.05 vs. the CN-W or CN-S offspring according to a Student’s *t*-test. Offspring were exposed to either an intrauterine environment of 20% (CN)-feeding mothers or that of 9% (LP)-Casein diet-feeding mothers, and provided with either water (W) or 1% saline drinking supply (S).

Renal *ptger1* expression levels were significantly higher in the LP-S and LP-W than in the CN-S and CN-W offspring, respectively (Figure 4a,b). Conversely, expression of the downstream epithelial Na^+^ channel alpha (*enacα*) gene was significantly decreased in LP-S compared to CN-S offspring (Figure 4d), but was unchanged between the CN-W and LP-W offspring (Figure 4c). The expression of additional genes down- and upstream of *ptger1*, comprising those that encode the beta (*enacβ*) and gamma (*enacγ*) epithelial Na^+^ channel subunits, and that which encodes prostaglandin E synthase 3 (*ptges3*), respectively, were also examined, and shown to be similar between the various offspring groups (SI Figure S2).

## 4. Discussion

This study is the first to examine the effect(s) of maternal protein restriction on DNA methylation and gene expression in SHRSP offspring. The elevation of the blood pressure and urinary albumin excretion in the 12-week-old LP-S offspring suggests that the salt sensitivity of the offspring was increased in response to the imposed low maternal protein consumption. Consistent with our previous reports, this effect did not impact the blood pressure, nor the urinary albumin excretion of the LP-W (compared to the CN-W) offspring [12,13].

From the results of the conducted methylome analysis, we selected *ptger1* for further analysis, due to its known association with hypertension [15]. As discussed, *ptger1* encodes the EP1 subtype of the PGE2 receptor, which is one of four PGE2 receptor subtypes (EP1–4) [16]. PGE2 is a critical product of arachidonic acid metabolism in the kidney, and functions to transport both Na^+^ and water, strain vascular smooth muscle, filter glomeruli, and promote renin secretion [17]. In fact, intrarenal PGE2 production has been shown to increase as RAAS activity levels rise [17]. Accordingly, a previous study demonstrated that (orally) administering 10-week-old SHRSP with a selective EP1 antagonist for five weeks was sufficient to cause both decreases in arteriole wounding, *transforming growth factor-beta* (*TGF-β*) expression, renal tubule stroma fibrosis, and urinary protein excretion, and an increase in renal creatinine clearance [15]. Thus, PGE2/EP1 signaling is thought to promote renal damage when blood pressure is elevated. Notably, our bisulfate analysis revealed that the *ptger1* methylation status was increased in the LP-S and LP-W compared to the CN-S and CN-W offspring, respectively (Figure 3). This suggests that the renal *ptger1* sequence was hypermethylated in offspring in response to the imposed maternal low-protein intake. In addition, the DNA methylation profile at the analyzed CpGs varied markedly between offspring provided with a saline compared to a normal drinking supply; thus, DNA methylation was affected by the imposed changes to both the maternal dietary protein, and offspring adult salt water intake.

While a number of studies have reported that gene transcription is inactivated by promoter methylation, others have shown it to cause gene expression to increase [18,19]. To date, the impact of methylation in transcribed regions on gene expression regulation is not well understood; however, some cases of upregulated gene expression resulting from hypermethylation within transcribed regions have been reported [20,21]. In this study, the *ptger1* DNA methylation profile in LP-W and LP-S offspring was increased by exposure to maternal protein restriction. The same offspring also exhibited significantly increased *ptger1* expression, suggesting that the incurred methylation changes may have upregulated *ptger1* mRNA expression. Thus, *ptger1* may be a novel epigenetic marker for salt sensitivity in the presence (or absence) of fetal protein restriction; however, further study is needed to confirm the effects of *ptger1* methylation and the effects of other important factors for expression of *ptger1*.

According to Guyton et al. [22], hypertension can occur when shifts in the relationship between renal pressure and natriuresis cause an increased salt intake, leading to high blood pressure. Thus, aberrant renal Na^+^ excretion is required for the development of salt sensitivity [22]. The epithelial Na^+^ channel (ENaC) critically regulates renal Na^+^ excretion, and thereby also salt sensitivity [23]. As discussed, there are three homologous ENaC subtypes, comprising the α (the major subtype), β, and γ subtypes [24], and ENaC activity is positively regulated by aldosterone [25]. In fact, *enacα* expression has been shown to increase in the presence of aldosterone, leading to enhanced Na^+^ resorption [26]; however, this is prevented by RAAS-induced PGE2/EP1 activation [27]. In the present study, the plasma aldosterone concentration was significantly increased in the LP-W offspring; however, this difference was not accompanied by a change in *enacα* expression levels. The increased *ptger1* mRNA expression in these offspring may have suppressed the effect of aldosterone on *enacα* mRNA induction, thereby preventing the expected aberrant renal Na^+^ excretion and blood pressure elevation. Conversely, the LP-S offspring exhibited reduced *enacα* expression, likely as a result of their observed increased *ptger1* expression, and unchanged plasma aldosterone concentration. Together, these observations suggest that reduced *enacα* mRNA levels suppress renal Na^+^ reabsorption, and that aberrant Na^+^ excretion likely mediated the salt sensitivity exhibited by the LP-S offspring.

As the future work of this study, we think that it will be important to reveal whether these *ptger1* and *enacα* changes are found in other rodent models such as normotensive model rat (e.g., Wistar rat and WKY rat) and mice models because it is unclear if our results are hypertensive model rat specific. In addition, we used kidney as the target organ for elucidation of a mechanism of hypertension, and it will be interesting to analyze other organs like the brain and heart, which we have already collected in this study. In consideration of investigating live humans (clinical tests), studies using accessible tissues like blood may be useful.

## 5. Conclusions

**Figure 5:**
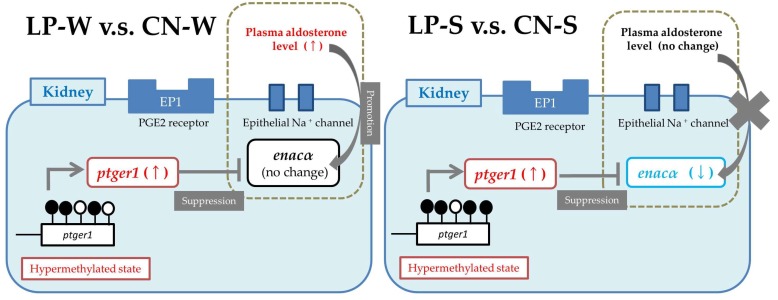
Schematic diagram showing the hypothesized changes to renal Na^+^ transport/homeostasis that were incurred in the Spontaneously Hypertensive Stroke Prone Rat (SHRSP) offspring by the imposed maternal protein restriction.

The results of the present study suggest that the imposed maternal protein restriction modulated renal *ptger1* methylation in the SHRSP offspring, and that the induced *ptger1* methylation changes likely altered renal *ptger1* and *enacα* gene expression to induce salt sensitivity (Figure 5). Further research is needed to confirm these conclusions, and to investigate whether the same mechanisms are important during human development.

## Figures and Tables

**Table 1:** Diet composition.

Component (%)	CN (20% Casein)	LP (9% Casein)
Casein	20.0	9.0
Corn starch	66.8	77.8
DL-Methionine	0.2	0.2
Soy bean oil	5.0	5.0
Vitamin mixture *	1.0	1.0
Mineral mixture *	4.0	4.0
Cellulose powder	3.0	3.0

* AIN-76 prescription (Oriental Yeast Co., Ltd., Tokyo, Japan). CN, 20%-Casein diet; LP, 9%-Casein diet.

## Supplementary Materials

The following are available online at http://www.mdpi.com/2072-6643/10/10/1436/s1, Table S1: Primers used in the real-time (RT)-PCR analysis of ptger1-related gene expression, Table S2: Liquid consumption, urine volume, and organ weights exhibited by offspring provided with drinking water (W), and either a high (CN) or low (LP)-protein diet, Table S3: Liquid consumption, urine volume, and organ weights in exhibited by offspring provided with a 1% saline drinking solution (S), and either a high (CN) or low (LP)-protein diet, Figure S1: ptger1 DNA methylation status according to the conducted bisulfite-sequencing analysis. CN, 20%-Casein diet; LP, 9%-Casein diet; S, 1% saline drinking solution; W, drinking water, Figure S2: Relative renal mRNA expression of ptger1-related genes. Values are expressed as the mean ± standard error (*n* = 7–8). CN, 20%-Casein diet; LP, 9%-Casein diet; S, 1% saline drinking solution; W, drinking water.
