# Supplementary material for: Maternal Protein Restriction Alters the Renal Ptger1 DNA Methylation State in SHRSP Offspring
Source: Nutrients. 2018 Oct 5;10(10):1436. doi: 10.3390/nu10101436 (PMC6213780; doi:10.3390/nu10101436)
Supplement: Supplementary file 1 [file nutrients-10-01436-s001.pdf]

Supplemental Information

Table S1. Primers used in the real-time(RT)-PCR analysis of *ptger1*-related gene expression.

Gene	Forward primer	Reverse primer
enacα	5’-AAGTGGACCGGAAGGACTGG-3’	5’-CCTGGACAGAATGTTGATGTAATGG-3’
enacβ	5’-TTTGCCTGCTGGGGAGA-3’	5’-GGTCATCTTATACTGGGTGTCGTTG-3’
enacγ	5’-GAAATGGTTGCTGAATGTTCTCAC-3’	5’-TACTGTTGGCTGGGCTCTCC-3’
ptger1	5’-AGTAGCTGGAGTGGGCAGCA-3’	5’-GCTCATATCAGTGGCCAAGAGG-3’
ptges3	5’-AGATTTACCAGAAGTAGACGGAGCA-3’	5’-CCAGGCGATGATACCACTCTTTAC-3’

Table S2. Liquid consumption, urine volume, and organ weights exhibited by offspring provided with drinking water (W), and either a high (CN) or low (LP)-protein diet.

Parameter	CN-W	LP-W
Drinking water consumption (ml/day)	25.3 ± 1.04	24.5 ± 1.49
Urine volume (ml/day)	10.4 ± 0.65	10.8 ± 0.30
Body weight (g)	301 ± 3.30	288 ± 5.82
Kidney weight (g)	2.48 ± 0.04	2.45 ± 0.05
Brain weight (g)	1.90 ± 0.02	1.86 ± 0.02
Heart weight (g)	1.15 ± 0.01	1.15 ± 0.02

Values are expressed as the mean ± standard error (n=7). CN, 20%-Casein diet; LP, 9%-Casein diet.

Table S3. Liquid consumption, urine volume, and organ weights in exhibited by offspring provided with a 1% saline drinking solution (S), and either a high (CN) or low (LP)-protein diet.

**Parameter**	**CN-S**	**LP-S**
Saline drinking solution consumption (ml/day)	41.4 ± 1.5	48.9 ± 3.0
Urine volume (ml/day)	20.8 ± 3.3	25.3 ± 4.8
Body weight (g)	255 ± 3.30	264 ± 7.93
Kidney weight (g)	2.00 ± 0.02	2.13 ± 0.15
Brain weight (g)	1.87 ± 0.01	1.90 ± 0.03
Heart weight (g)	1.03 ± 0.02	1.11 ± 0.07*

Values are expressed as means ± standard error (n=7-8). *$p < 0.05$ vs. the CN-S group according to a Student's t-test.).

CN, 20%-Casein diet; LP, 9%-Casein diet.

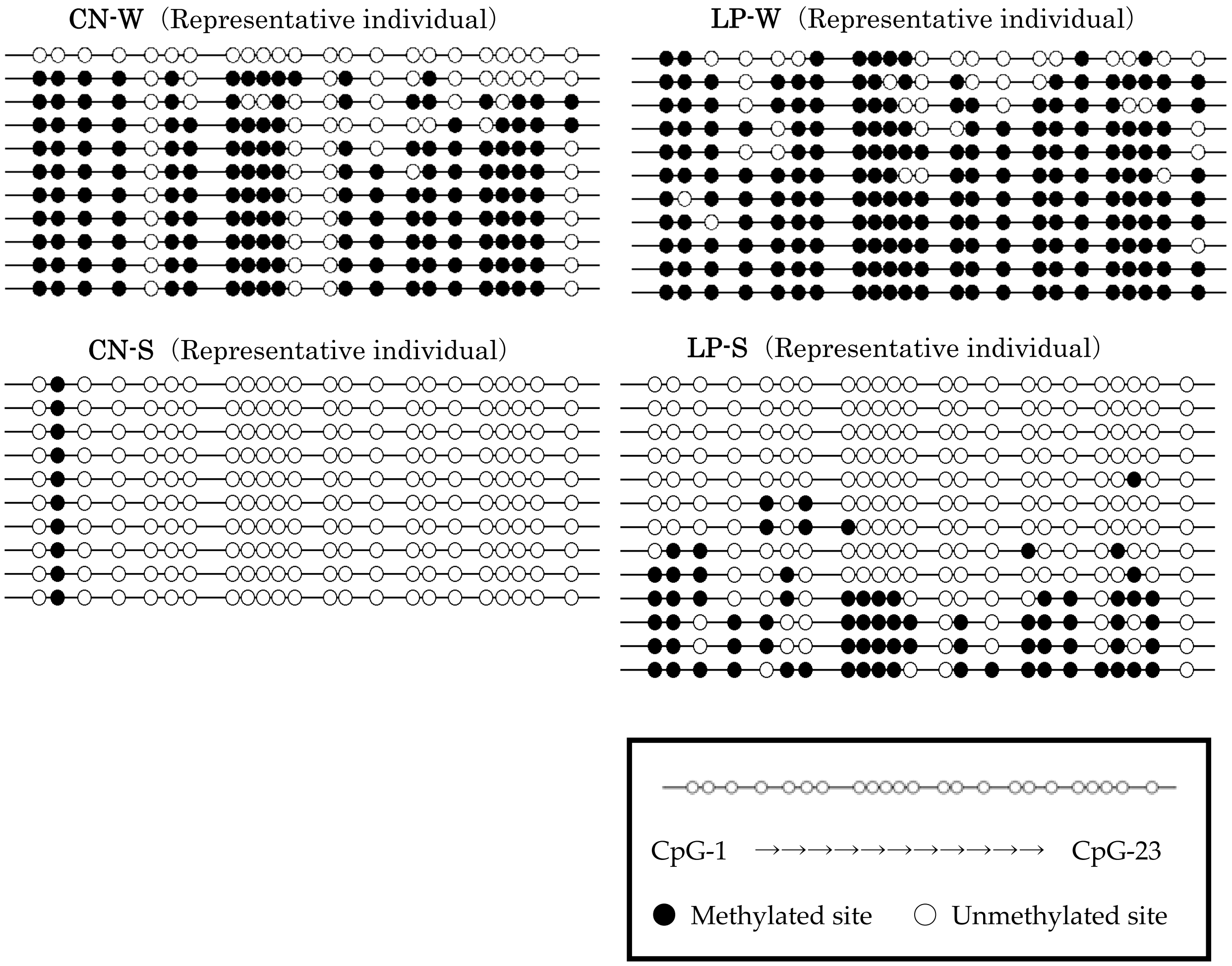

Figure S1. *ptger1* DNA methylation status according to the conducted bisulfite-sequencing analysis. CN, 20%-Casein diet; LP, 9%-Casein diet; S, 1% saline drinking solution; W, drinking water.

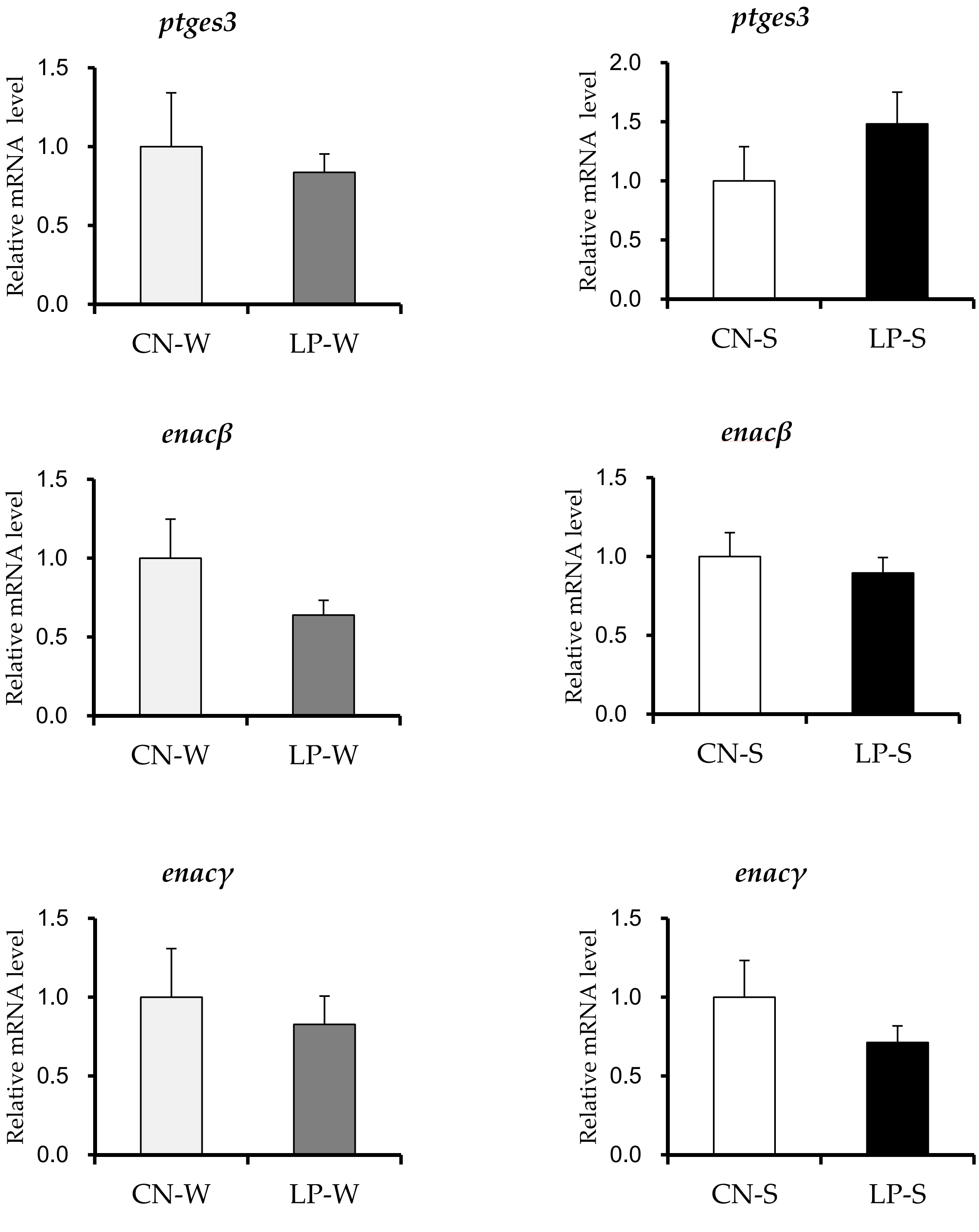

Figure S2. Relative renal mRNA expression of *ptger1*-related genes. Values are expressed as the mean ± standard error (n=7–8). CN, 20%-Casein diet; LP, 9%-Casein diet; S, 1% saline drinking solution; W, drinking water.
